# Structural control of corneal transparency, refractive power and dynamics

**DOI:** 10.1038/s41433-024-02969-7

**Published:** 2024-02-23

**Authors:** Keith M. Meek, Carlo Knupp, Philip N. Lewis, Siân R. Morgan, Sally Hayes

**Affiliations:** Structural Biophysics Group, School of Optometry and Vision Sciences, Cardiff University, Maindy Road, Cardiff, CF24 4HQ UK

**Keywords:** Anatomy, Biological techniques

## Abstract

The cornea needs to be transparent to visible light and precisely curved to provide the correct refractive power. Both properties are governed by its structure. Corneal transparency arises from constructive interference of visible light due to the relatively ordered arrangement of collagen fibrils in the corneal stroma. The arrangement is controlled by the negatively charged proteoglycans surrounding the fibrils. Small changes in fibril organisation can be tolerated but larger changes cause light scattering. Corneal keratocytes do not scatter light because their refractive index matches that of the surrounding matrix. When activated, however, they become fibroblasts that have a lower refractive index. Modelling shows that this change in refractive index significantly increases light scatter. At the microscopic level, the corneal stroma has a lamellar structure, the parallel collagen fibrils within each lamella making a large angle with those of adjacent lamellae. X-ray scattering has shown that the lamellae have preferred orientations in the human cornea: inferior-superior and nasal-temporal in the central cornea and circumferential at the limbus. The directions at the centre of the cornea may help withstand the pull of the extraocular muscles whereas the pseudo-circular arrangement at the limbus supports the change in curvature between the cornea and sclera. Elastic fibres are also present; in the limbus they contain fibrillin microfibrils surrounding an elastin core, whereas at the centre of the cornea, they exist as thin bundles of fibrillin-rich microfibrils. We present a model based on the structure described above that may explain how the cornea withstands repeated pressure changes due to the ocular pulse.

## Abstract (zh)

角膜对可见光透明且具有精确的曲率, 以提供正确的折射能力。这两个特性都受其结构调控。角膜透明性是由于角膜基质中胶原纤维的相对有序排列, 使得可见光产生结构性干涉而形成的。这种排列受胶原纤维周围的带负电荷的蛋白多糖控制。对纤维组织的微小变化可耐受, 但较大的改变会导致光散射。角膜角质细胞不会使光散射, 因为它们的折射率与周围基质一致。但是, 当被激活时, 它们会变成折射率较低的成纤维细胞。建模显示, 这种变化会显著增加光的散射。在微观层面上, 角膜基质呈层状结构, 每层中的平行胶原纤维与其相邻层的纤维呈较大角度分布。X射线散射显示, 在人类角膜中这些片层具有优选方向: 角膜中央为上下、鼻-颞方向, 角膜缘为环形分布。角膜中央方向有助于抵御眼外肌的牵引, 而角膜缘处的伪圆形排列支持角膜与巩膜之间曲率的变化。弹性纤维也存在；在角膜缘, 它们含有围绕弹性蛋白核心的原纤维蛋白微纤维, 而在角膜中心, 它们以富含纤维蛋白的微纤维束的形式存在。我们提出了一个基于上述结构的模型, 该模型可以解释角膜如何乘受由于眼动脉搏动导致的反复压力变化。

## Introduction

The cornea is the primary lens in the eye, responsible for about two-thirds of the refractive power. Most of this refraction takes place between the air and the tear film that covers the corneal surface and is a result of the difference in refractive index between these two media. Like any convex lens, the surface power *D* in air (and hence the focal length) is given by:

*D* = (*µ*−1)/*r*, where *µ* is the refractive index and *r* is the radius of curvature. Thus, for any lens to function properly, it is important that it has a smooth front surface with a well-defined radius of curvature. The average radius of curvature of the corneal surface is 7.79 ± 0.27 mm [1], but the surface is flatter horizontally than vertically. Although the refractive index of the cornea/tear film is depth-dependent and many different values appear in the literature [2], most researchers use 1.376 as a working average value for the corneal stroma [2, 3].

Of course, it is also necessary that a lens is transparent to the light passing through it. In the case of the normal cornea, over 90% of visible light is transmitted, but this drops off rapidly at the ultraviolet end of the spectrum [4]. There are no molecules that absorb visible light and the small loss in transmittance is mostly due to scattering by cell nuclei [5].

The corneal stroma makes up about 90% of the total tissue thickness. Anterior to the stroma are Bowman’s layer and the epithelium, posterior are Descemet’s membrane and the endothelium. The stroma is not only responsible for the strength of the cornea as part of the ocular tunic, but also for the maintenance of both transparency and corneal curvature. In this paper we detail our current understanding of the structure of the corneal stroma. We show that both the nanostructure and the microstructure are dynamic systems and go on to describe how these different hierarchical structures promote and maintain both transparency and correct refractive power.

### Corneal nanostructure and transparency

**Fig. 1:**
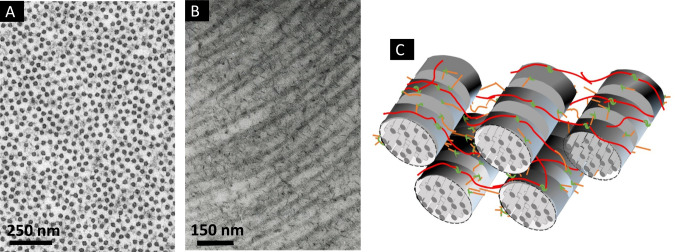
Arrangement of collagen and proteoglycans in the human cornea. **A** Transmission electron micrograph of corneal collagen fibrils in cross-section stained with phosphotungstic acid and uranyl acetate. **B** Longitudinal section of corneal collagen fibrils counterstained with cuprolinic blue to visualise proteoglycans. **C** The collagen and proteoglycan organisation in the corneal stroma. Fibrils contain collagen molecules staggered axially to produce a periodic banding along the fibril axis. The protein cores of the proteoglycans (green) are associated with the fibrils at specific binding sites. Attached to the protein cores are either keratan sulphate glycosaminoglycans (orange) or dermatan/chondroitin sulphate glycosaminoglycans (red). **A**, **B** supplied by Dr Rob Young and Prof Andrew Quantock (Cardiff University) and (**C**) reproduced from Meek and Hayes [39].

The principal protein in the cornea is collagen, which exists in the form of narrow uniform-diameter fibrils about 30 nm wide [6]. Each fibril contains ∼400 tropocollagen molecules and the fibrils are parallel to one another within lamellae. Figure 1A shows that in transverse section, fibrils in the healthy corneal stroma pack together with a degree of short-range order. This order is maintained by the presence of proteoglycan molecules between the fibrils (Fig. 1B). There are two types of proteoglycan in the stroma, one containing keratan sulphate glycosaminoglycans and the other containing dermatan/chondroitin sulphate glycosaminoglycans. Each proteoglycan links to the collagen at a specific binding site along the fibril axis via a core protein. While the shorter keratan sulphate glycosaminoglycans tend to coat the fibrils, the longer dermatan/chondroitin sulphate glycosaminoglycans form dimers that can bridge several collagen fibrils (Fig. 1C).

Both types of proteoglycan are highly negatively charged. If adjacent fibrils approach one another, the negative charge density increases, and this attracts additional counterions and water (via the Gibbs-Donnan effect) that push the fibrils apart, extending the proteoglycan bridging dimers. This leads to a dynamic local movement of neighbouring fibrils (Supplementary Video 1). We have modelled the fibril kinetics by simulating the effects of thermal motion on each fibril, along with the attractive medium range forces between fibrils due to the action of the proteoglycans and the short-range repulsive forces due to the physical bulk of the collagen fibrils and surrounding water molecules. Supplementary Video 2 shows that, although this is a dynamic system, short-range order is maintained and, in a snapshot the structure resembles that seen in electron micrographs such as Fig. 1A.

**Fig. 2:**
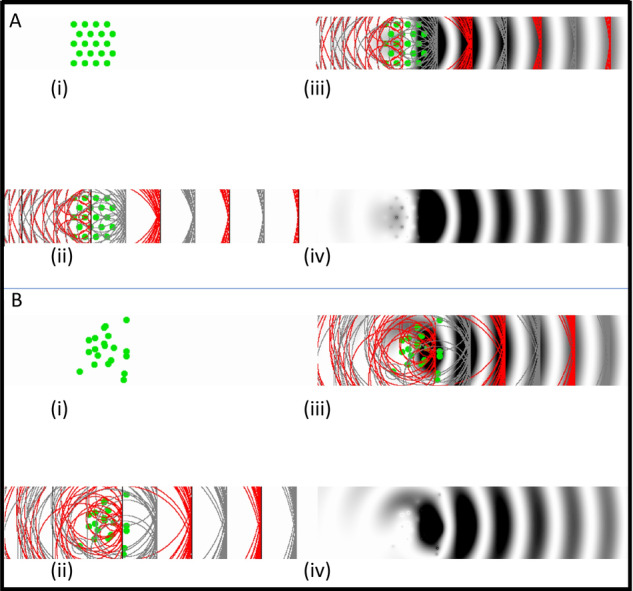
Light scattering from corneal collagen fibrils. **A** (i) Each fibril in a regular array of collagen fibrils (green) will scatter parallel wavefronts incident from the left in all directions (only the forward and backward directions are shown in ii and iii). (ii) The secondary wavefronts (red and grey circles) interfere constructively in the forward direction and destructively in other directions. (iii) These wavefronts are shown superimposed on the full intensity field. (iv) The intensity field of forward and backscattered radiation. **B** (i) A disordered array of fibrils. (ii) The secondary wavefronts from this array, (iii) The secondary wavefronts superimposed on the full intensity field, showing significant constructive interference in the backward direction in addition to the forward direction. (iv) The intensity field of forward and backscattered radiation from the disordered array of collagen. Animated versions of this figure are presented in Supplementary Videos 3 and 4.

When light impinges on this array of collagen fibrils, Rayleigh scatter occurs and each fibril scatters the light in all radial directions, but because of the short-range order, there is constructive interference of all the scattered waves in the forward direction and mostly destructive interference in other directions (Fig. 2A, Supplementary Video 3). However, if sufficient local disorder is introduced into the array, a significant amount of the light is scattered (Fig. 2B, Supplementary Video 4), and if voids in the structure exceed half the wavelength of light, the scattering increases dramatically. This is the cause of the cloudiness seen in corneal oedema, where the incoming fluid tends to aggregate in ‘lakes’ within the stroma. A detailed explanation of corneal transparency can be found in Meek and Knupp [4].

**Fig. 3:**
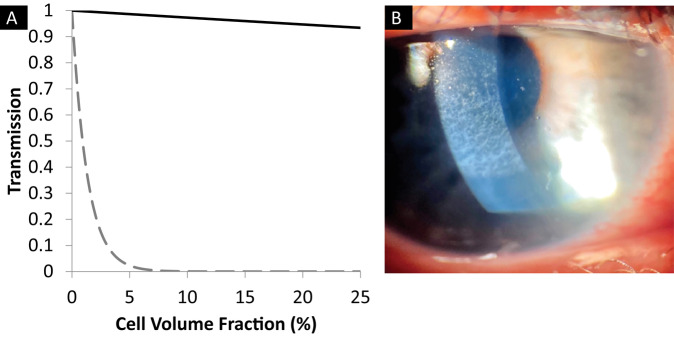
Light scattering from cells in the corneal stroma. **A** Predicted transmission (based on modelling of Mie scatter) from the anterior cornea uniformly populated with keratocytes (solid line) or fibroblasts (broken line) [9]. **B** Anterior corneal haze following photorefractive keratectomy (image reproduced with permission from https://crstodayeurope.com/articles/july-august-2021/corneal-haze-after-prk-enhancement-of-prior-lasik/).

In addition to collagen, the stroma contains many large cells called keratocytes. A question, which long puzzled scientists, was why do these cells not scatter the light? It was originally believed that, because the cells are flat and sparse, they do not contribute significantly to light scatter. However, later work showed that the keratocytes in fact constitute up to 17% of the stromal volume [7]. To explain cell transparency, Jester et al. [8] suggested that the corneal cells, similarly to crystalline lens cells, preferentially express water-soluble proteins, transketolase and aldehyde dehydrogenase 1. These are thought to match the refractive index of the cells to that of the rest of the stroma, thus eliminating light scatter at the matrix-cell boundaries, and the authors provided substantial evidence to support this suggestion [7]. Accordingly, we used a technique called quantitative phase imaging to measure the refractive index of the keratocytes and showed that they have a refractive index of 1.381 ± 0.004 [9]. This closely matches the refractive index of the stroma as a whole and appears to confirm Jester et al.’s explanation for cellular transparency. We also measured how the refractive index of the cells changes when they are activated to become fibroblasts, such as during wound healing, discovering that the refractive index of the cytoplasm dropped to 1.365 ± 0.003. Scatter from the relatively large cells (compared with the wavelength of light) is termed Mie scatter. We modelled this Mie scatter by treating the cells as discs of known dimensions and refractive index. The results are shown in Fig. 3A. It can be seen that, as the proportion of keratocytes increases, there is very little loss of transparency, but the opposite is true for fibroblasts [9]. We previously showed that haze following laser refractive ablation is not caused by the deposition of fibrotic tissue [10]. Our cell refractive index measurements show that such haze (Fig. 3B) results from the presence of fibroblasts in the ablated region.

### Corneal microstructure and refractive power

**Fig. 4:**
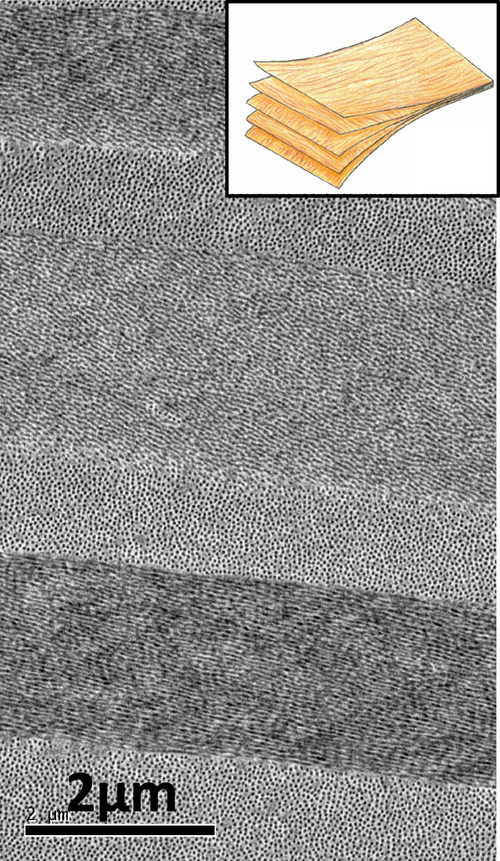
Organisation of corneal lamellae. As observed by electron microscopy the arrangement of posterior stromal lamellae may be likened to the structure of plywood (inset) where the grain runs in different directions in adjacent layers.

As part of the eyeball, the cornea needs to be strong to withstand the intraocular pressure (IOP). This strength is provided by the microstructure of the stroma, whereby fibrils are arranged within lamellae. In the posterior two-thirds of the stroma, the lamellae lie one above the other, seemingly running from limbus to limbus. Along the direction of the fibrils within a lamella, collagen is as strong as steel, and the presence of fibrils in all directions parallel to its surface gives the tissue the necessary high tensile strength within the corneal plane. In addition, the lamellar structure acts rather like plywood, which resists fracture propagation, but bends and returns to its original position [11] (Fig. 4).

**Fig. 5:**
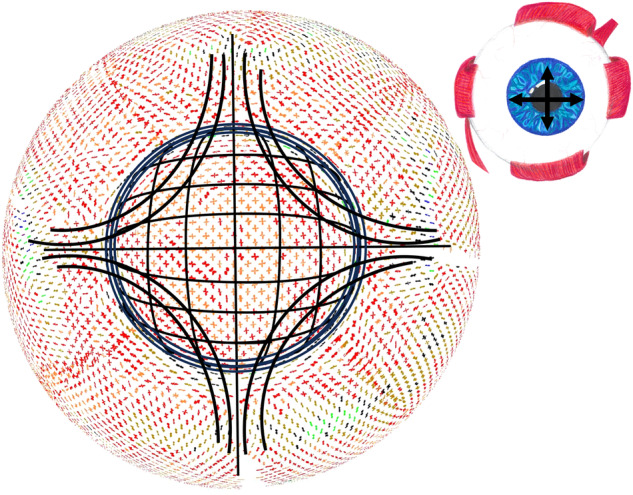
Predominant collagen orientations in the cornea and sclera. X-ray scatter data shows the preferred directions of collagen lamellae in different parts of the cornea and sclera (courtesy of Dr. Craig Boote, Cardiff University). Superimposed is our simplified model of the proposed overall preferential directions of lamellae, based on the x-ray data [13]. These preferential directions are believed to help support the curvature of the cornea and maintain its shape under the pull of the extraocular muscles (inset).

X-ray scattering has allowed us to investigate if, over and above the uniform radial distribution of collagen lamellae, there exist preferred orientations to reinforce the tissue in certain directions. The two-dimensional x-ray-derived collagen orientation map of the cornea and sclera [12] is shown in Fig. 5, upon which is superimposed our model for the preferential directions of lamellae inferred from similar data [13]. The map shows a distinct annulus of collagen at the limbus [14] located in the deeper limbal stroma [15], which is required to maintain the change in curvature between the cornea and adjacent sclera [16]. Throughout the optical zone, there is a preference for lamellae to run in the inferior-superior and temporal-nasal directions. Many of these appear to continue into the sclera and are believed to help the cornea to withstand the pull of the extraocular muscles [17–19] (Fig. 5 inset).

**Fig. 6:**
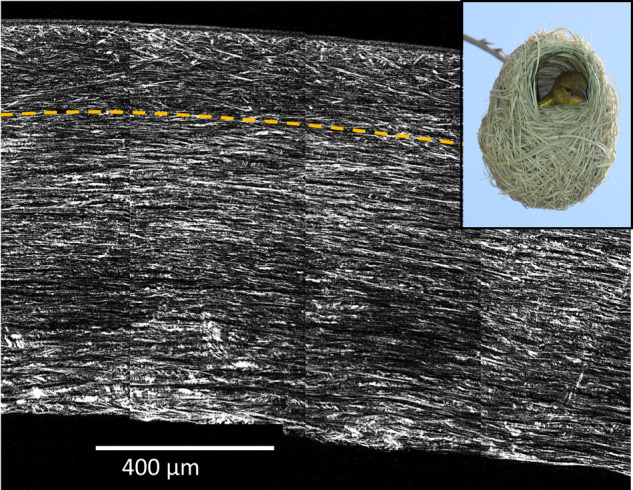
Anterior lamellar interweaving. Second harmonic generated composite image showing lamellar interweaving in the anterior stroma above the dashed line [40]. The stromal interweaving supports corneal curvature similar to interweaving of fibres in a bird’s nest (inset image reproduced with permission by Neerav Bhatt via Flickr) (www.flickr.com/photos/neeravbhatt/https://).

The curvature of the anterior cornea is crucial for it to provide the correct refractive power, and this is maintained by lamella interweaving. Anterior lamellae, rather than being stacked one on top of another, often split and are highly interwoven [20]. This arrangement is analogous to a bird’s nest and has been described as a bow spring-like structure [21] where the lamellae seem to be anchored in Bowman’s layer (Fig. 6).

### Maintenance of refractive power in vivo

**Fig. 7:**
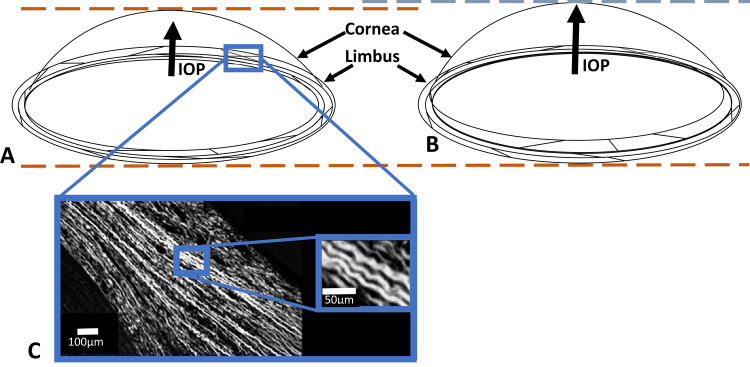
Possible response of the cornea to fluctuations of IOP. **A**, **B** Model showing how the cornea is displaced forward when the IOP is increased. The shape of the cornea remains the same and the displacement occurs in the limbal region where the annular meshwork of collagen lamellae opens. This displacement would necessitate stretching of any interconnecting lamellae. **C** We propose that this stretching is facilitated by the uncrimping of the crimped collagen in the limbus (image reproduced with permission from [27]). An animated version of this model is shown in Supplementary video 5.

In vivo, the blood flow through the choroid leads to a fluctuation of the IOP known as the ocular pulse that has an amplitude of about 2 mmHg [22, 23]. Boyce et al. [24] showed that, when pressure within the bovine eye is increased, the apex of the cornea moves forward the same amount as the mid periphery; in other words, the central cornea is displaced forward as a whole thereby maintaining its shape, and the stretching takes place in the peripheral cornea and limbus. Based on these results we proposed a model using our structural results to explain these dynamics (Fig. 7).

The model shows that, when the IOP increases, the central cornea moves forward whilst retaining its shape (Fig. 7B). The limbal annulus pushes forward, stretching any lamellae that criss-cross and hold the structure together. Direct experimental evidence for this model was provided by Wilson et al. [25], who showed using interferometry that with small IOP increments (0.25 mmHg) the highest rate of change of the out-of-plane displacement takes place at the limbus. Furthermore, Elsheikh et al. [26] showed that the average stiffness of the human cornea is constant across the central optical zone then falls off rapidly towards the limbus. Supplementary Video 5 shows an animation of this model representing the dynamics of the system as the IOP pulses.

For the above model to work, it would be necessary for any interconnecting lamellae within the limbal annulus, as well as radial lamellae anterior to the annulus, to stretch when the cornea moves forward. As in most collagenous tissues, corneal lamellae, including those in the limbus, are known to have a natural waviness called crimp [27] (Fig. 7C), and this could provide a mechanism to allow a lamella to elongate without injury [28]. However, the underlying structural cause of this crimp is not fully understood, and the probability is that repetitive stretching during a lifetime would condition the tissue and remove the crimp. The restoring force in those connective tissues that are required to repetitively stretch and return to their original form, such as skin or heart valve, is provided by elastic fibres. Consequently, we developed a staining technique using serial block face scanning electron microscopy (SBF SEM) to detect elastic fibres and their precursors, and to follow their course through the corneal stroma [29, 30].

**Fig. 8:**
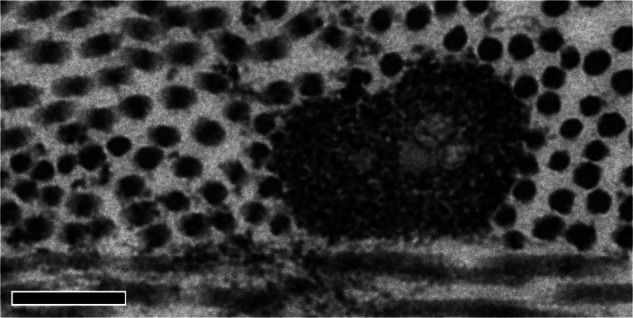
Transmission electron micrograph of an elastic fibre [29]. The elastic fibre in transverse section shows an amorphous elastin core surrounded by fibrillin-rich microfibril bundles. Scale bar: 200 nm.

**Fig. 9:**
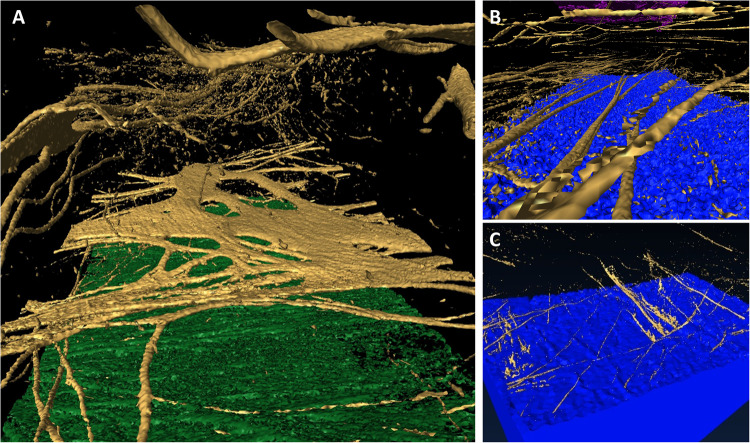
SBF SEM rendered images showing the three-dimensional arrangement of elastic fibres in different regions of the human cornea. **A** The limbus (limbal elastic sheets/fibres are rendered in gold and the trabecular elastic fibres are rendered in green) showing reticulated elastic fibre sheets. **B** The peripheral cornea (elastic fibres are rendered in gold and Descemet’s membrane is coloured blue) showing elastic fibres dividing as they progress towards the centre of the cornea. **C** Narrow fibrillin-rich microfibril bundles in the centre of the human cornea (microfibril bundles rendered in gold, Descemet’s membrane coloured blue).

Such fibres consist of an outer shell of fibrillin-rich microfibrils with or without an elastin central core (Fig. 8). The presence of elastic fibres within corneas has been known for a long time, but it was unclear whether or not they exist in the mature human cornea [31, 32]. Our SBF SEM observations revealed that, concentrated at the back of the human corneal stroma above the elastin-rich trabecular meshwork, is a region densely populated with elastic fibres (Fig. 9A) arranged in a reticulated sheet pattern [29]. In the peripheral cornea, emanating from these sheets, we found elastic fibres that bifurcated or trifurcated into narrower fibres (Fig. 9B) concentrated in the region corresponding to the pre-Descemet’s layer reported by Dua et al. [33]. At the centre of the cornea were mostly narrow fibres (Fig. 9) the majority of which had no elastin core so are assumed to be bundles of fibrillin microfibrils. It has been suggested that these microfibrils play a role in maintaining corneal shape [34, 35].

**Fig. 10:**
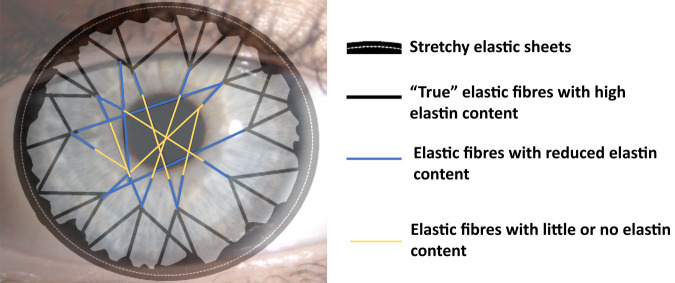
The proposed elastic fibre network in the cornea. Elastic fibre sheets are associated with the pseudo-circumferential limbal elastic fibres. From these, ‘true’ elastic fibres traverse the peripheral cornea. These split into narrower bundles of fibrillin-rich microfibrils as they approach the centre of the cornea, where most have lost their elastin core [36].

Figure 10 shows the postulated arrangement of elastic fibres based on these SBF SEM results [36]. It should be stressed that we have not been able to follow individual fibres over long distances and have therefore assumed that they traverse the whole cornea from limbus to limbus. This seems probable as we have not noticed any obvious fibre ends, nor any anchoring points within the matrix. The model shows how, as they move centrally, most of the fibres gradually lose their elastin and become bundles of fibrillin microfibrils. The properties of these microfibrils are complex as they depend on what other molecules are associated with the fibrillin, the amount of cross-linking etc. [37]. However, although they can extend, they are not intrinsically elastic and their Young’s modulus is two orders of magnitude higher than that of elastin [38].

**Fig. 11:**
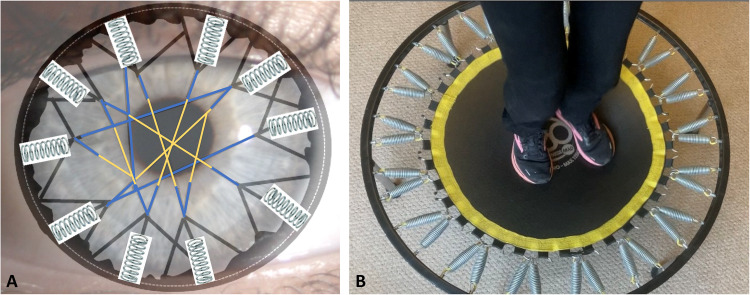
Model to explain how the elastic sheets and elastin-rich fibres in the limbal and peripheral stroma may provide the force required to restore the cornea to its normal position during the ocular pulse. The elastic fibres are proposed to act like springs (**A**) with a function analogous to the springs in a trampoline (**B**).

Such an arrangement of elastic fibres suggests a possible mechanism that provides the restoring force at the limbus required to sustain the repetitive effects of the ocular pulse (Fig. 11). The ‘true’ elastic fibre sheets in the limbal region act like the springs of a trampoline, storing the potential energy as the cornea is pushed forward by the increased IOP, and releasing it when the IOP reduces. The connecting fibrillin microfibrils in the central cornea are less extensible and this, combined with the interweaving of the central anterior lamellae described above, limits distortion of the central cornea. We have therefore termed this the ‘trampoline effect’. It is worth noting that the central keratoconus cornea has very few elastic fibres or fibrillin microfibrils [36]. It would therefore be of some interest to discover if the ‘true’ elastic fibres are also reduced in the keratoconus limbus. If this were the case, there would be either no mechanism or an impaired mechanism to absorb the structural changes associated with the ocular pulse, which could, in the long term, contribute to the observed tissue failure. The elastic movement in the limbus due to the ocular pulse may also occur in the underlying trabecular meshwork, particularly if the elastic fibres in these two regions are linked. This could be of some importance as it would provide a natural pumping mechanism in the trabecular meshwork driven by the ocular pulse.

In conclusion, the cornea is a dynamic structure at all hierarchical levels. Vibrational motion of fibrils at the nanoscopic level is controlled by the negative charges on the surrounding glycosaminoglycans which maintain sufficient collagen fibril order to allow destructive interference of light in all directions other than forwards. We presented a model based on the proposed collagen and elastic fibre arrangements that may explain at the microscopic level how the corneal anterior displacement due to the ocular pulse occurs and how the tissue survives the continuous IOP changes during a lifetime.

## Supplementary information

Supplementary video 1

Supplementary video 2

Supplementary video 3

Supplementary video 4

Supplementary video 5

## Data Availability

All the data supporting the findings of this study are available within the article.
