# Supplementary figures and images for: Structural control of corneal transparency, refractive power and dynamics
Source: Eye (Lond). 2024 Feb 23;39(4):644–50. doi: 10.1038/s41433-024-02969-7 (PMC11885422; doi:10.1038/s41433-024-02969-7)

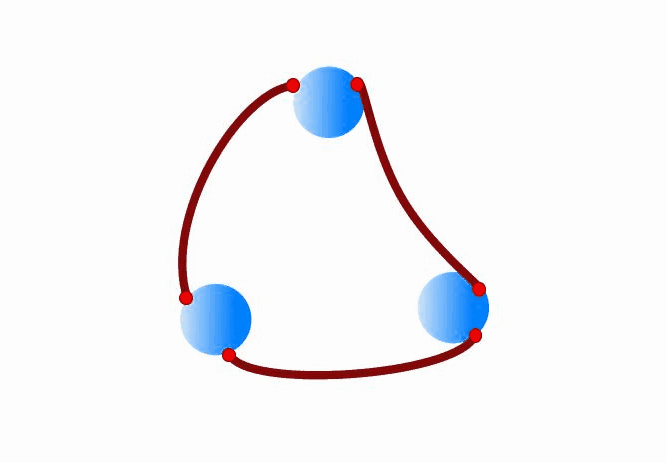

Supplement: Supplementary file 1 — Supplementary video 1 [file 41433_2024_2969_MOESM1_ESM.gif]

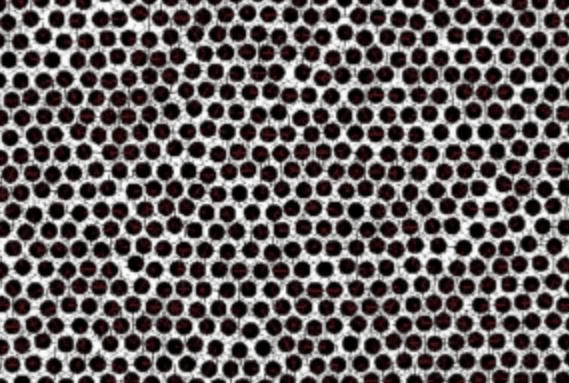

Supplement: Supplementary file 2 — Supplementary video 2 [file 41433_2024_2969_MOESM2_ESM.gif]

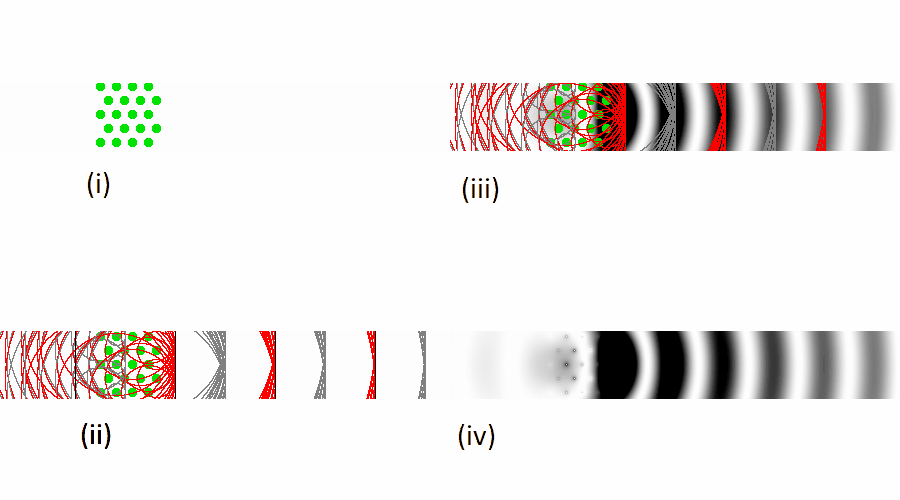

Supplement: Supplementary file 3 — Supplementary video 3 [file 41433_2024_2969_MOESM3_ESM.gif]

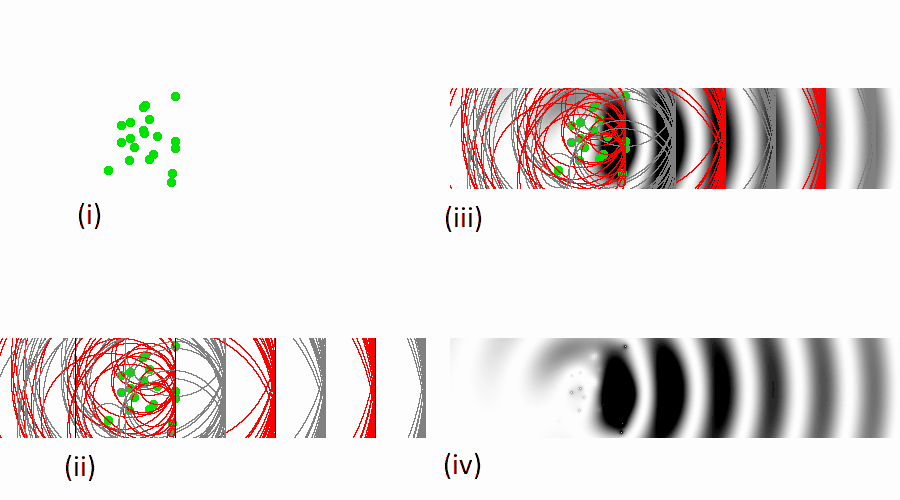

Supplement: Supplementary file 4 — Supplementary video 4 [file 41433_2024_2969_MOESM4_ESM.gif]
